# Mouse KL2 is a unique MTSE involved in chromosome-based spindle organization and regulated by multiple kinases during female meiosis

**DOI:** 10.7555/JBR.37.20230290

**Published:** 2024-05-29

**Authors:** Shiya Xie, Yanjie Yang, Zhen Jin, Xiaocong Liu, Shuping Zhang, Ning Su, Jiaqi Liu, Congrong Li, Dong Zhang, Leilei Gao, Zhixia Yang

**Affiliations:** 1 State Key Lab of Reproductive Medicine and Offspring Health, Nanjing Medical University, Nanjing, Jiangsu 211166, China; 2 Central Laboratory, the First Affiliated Hospital of Anhui Medical University, Hefei, Anhui 230022, China; 3 Department of Gynaecology and Obstetrics, the First Affiliated Hospital of Anhui Medical University, Hefei, Anhui 230022, China; 4 Center for Reproductive Medicine, Department of Gynecology, Zhejiang Provincial People's Hospital (Affiliated People's Hospital), Hangzhou Medical College, Hangzhou, Zhejiang 310014, China; 5 Center for Reproductive Medicine, Department of Reproductive Endocrinology, Zhejiang Provincial People's Hospital (Affiliated People's Hospital), Hangzhou Medical College, Hangzhou, Zhejiang 310014, China; 6 Laboratory Department of Shihezi People's Hospital, Shihezi, Xinjiang 832099, China

**Keywords:** mouse, KL2, MTSE, kinase, female meiosis

## Abstract

Microtubule-severing enzymes (MTSEs) play important roles in mitosis and meiosis of the primitive organisms. However, their roles in mammalian female meiosis, which accounts for over 80% of gamete-originated human reproductive diseases, remain unexplored. In the current study, we reported that katanin-like 2 (KL2) was the only MTSE concentrating at chromosomes. Furthermore, the knockdown of *KL2* significantly reduced the chromosome-based increase in the microtubule (MT) polymer, increased aberrant kinetochore-MT (K-MT) attachment, delayed meiosis, and severely affected normal fertility. We demonstrated that the inhibition of aurora B, a key kinase for correcting aberrant K-MT attachment, significantly eliminated KL2 expression from chromosomes. Additionally, KL2 interacted with phosphorylated eukaryotic elongation factor-2 kinase, and they competed for chromosome binding. Phosphorylated KL2 was also localized at spindle poles, with its phosphorylation regulated by extracellular signal-regulated kinase 1/2. In summary, the current study reveals a novel function of MTSEs in mammalian female meiosis and demonstrates that multiple kinases coordinate to regulate the levels of KL2 at chromosomes.

## Introduction

Microtubules (MTs) are a major class of the cytoskeleton in cells, and they play a key role in cell division, morphogenesis, motility, protein and organelle transport, signaling, and multiple neuronal activities. In mitosis, MTs form a large molecular structure known as the mitotic spindle that precisely separates chromosomes between two daughter cells. The MT polymer quickly rotates within the mitotic spindle and has a half-life of only a few minutes. The behavior of MTs, whether acting individually or in random harmony with other MTs in the spindle, depends on their path to the chromatin, their contact with the MT complex, spatial distribution of the organizing center, and centriole assembly ^[ 1– 3] ^. For example, one MT may be captured or released from the center without significantly affecting other MTs nearby. Additionally, MT groups attached to the center may cooperatively combine and detach to promote chromosome movement ^[ 4] ^. To perform these functions, MTs must remain highly dynamic *via* a well-balanced process of assembly and disassembly ^[ 1– 4] ^. This process is regulated by numerous proteins ^[ 1– 4] ^, including MT-severing proteins (MTSEs) ^[ 5– 9] ^. MTSEs belong to the "meiotic" subfamily of ATPases associated with diverse cellular activity (AAA) super family ^[ 10– 11] ^. All MTSEs have an AAA domain that binds to and hydrolyzes ATP to sever an MT along its length ^[ 10– 13] ^. Thus, the MT severing may be a more efficient way to reorganize MTs than the end-limited MT depolymerization by kinesins ^[ 14– 19] ^.

Mitosis is the key process for eukaryotic cell proliferation. The centrosome exists in most animal cells and serves as the primary MT-organizing center, ensuring the correct segregation of chromosomes during cell division. As the cell transitions from interphase to mitosis, the duplicated centrosomes separate and move to opposite sides of the cell, where the spindle apparatus assembles. The centrosome not only forms the nucleus but also organizes the MTs of the mitotic spindle. Proper orientation of the mitotic spindle is crucial for the correct division of the cell ^[ 20– 21] ^. Abnormal chromosome segregation may cause aneuploidy, which is one of the major causes of tumorigenesis ^[ 22] ^.

An increasing number of studies have shown that MTSEs play important roles in mitosis through their MT-severing activity. Katanin 60, the first identified MTSE ^[ 23] ^, is targeted to the centrosome ^[ 24]^ and facilitates γ-tubulin redistribution in human cells ^[ 25] ^. During female meiosis in *Caenorhabditis elegans*, the katanin 60 homolog MEI-1 is required for the translocation of the meiosis Ⅰ spindle to the oocyte cortex and for parallel/antiparallel MT organization in meiotic spindles ^[ 26] ^. Katanin activity is an important determinant of spindle length in *Xenopus tropicalis* and *X. laevis* egg extracts ^[ 27] ^. Katanin-like 2 (KL2) is a versatile enzyme that plays a significant role in processes affecting cell division, MT dynamics, and ciliogenesis, which is also associated with cell cycle progression. Furthermore, outside the centrosome, delta- and epsilon-tubulin proteins are located in the manchette structure during spermiogenesis in mice and interact with KL2, indicating a novel non-centrosomal function ^[ 28– 29] ^. The second identified MTSE member, spastin, was found to be mutated in the most frequent form of autosomal dominant spastic paraplegia ^[ 27] ^. Spastin was later shown to be an MTSE, and its centrosomal location demonstrated its centrosome-based mitotic function ^[ 5, 30– 31] ^. Fidgetin ^[ 7, 30] ^, fidgetin-like 1 ^[ 32] ^, katanin-like 1 (KL1) ^[ 33] ^, and KL2 ^[ 34]^ are also MTSEs, which have been reported to play crucial roles in mitosis. Their mitotic functions appear to be divergent, since they show different localization patterns within the cell, and a loss of function diversely affects MT dynamics and organization within the spindle.

Normal meiosis, especially female meiosis that involves two rounds of cell division, requires delicate control and is crucial for maintaining genome stability. Dysfunctional female meiosis is a major cause of human genetic diseases ^[ 35– 37] ^. The spindle in oocytes differs from the mitotic spindle in some key aspects, and the spindle that forms in the oocytes of many animals lacks a centrosome. Meiosis produces four daughter haploid cells from one diploid oocyte. To achieve successful asymmetric division, the spindle must be located near and perpendicular to the cellular cortex. Chromosome-MT interactions in oocytes may differ from those in mitosis. In mitosis, the primary interaction is provided by kinetosomes that interact with the ends of dynamic MTs, and non-kinetosomal interactions appear to be more prominent in oocytes than in mitotic cells. Spindle assembly checkpoints are the mechanisms that ensure proper chromosome separation and are critical for genomic stability. However, spindle assembly checkpoints in meiosis are not as robust as in mitosis, leading to a high incidence of chromosome separation errors in oocytes ^[ 38– 39] ^. Little is known about the role of MTSEs in mammalian female meiosis.

In the current study, we reported that KL2 was the only MTSE to be localized at chromosomes and important for chromosome-based spindle organization and meiosis through the coordinate regulation of multiple key kinases.

## Material and methods

### General chemicals and reagents and animals

Unless otherwise stated, all chemicals and reagents were sourced from Sigma (St. Louis, MO, USA). The Institute of Cancer Research (ICR) mice used in the current study were from Beijing Vital River Laboratory Animal Technology Co., Ltd. All animal experiments were approved by the Animal Ethical and Welfare Committee of Nanjing Medical University and conducted according to institutional guidelines (Approval No. IACUC-1903028).

### Antibodies and kinase inhibitors

Primary antibodies of mouse monoclonal anti-β-tubulin (Cat. #sc-5274, 1∶500), rabbit polyclonal anti-katanin p60 AL2 (N-15) (Cat. #sc-84855, 1∶1000),goat polyclonal anti-katanin p60 A1 (M-13) (Cat. #sc-109299, 1∶1000), mouse monoclonal anti-katanin p60 AL1 (A-10) (Cat. #sc-373814, 1∶500), rabbit polyclonal anti-fidgetin (H-146) (Cat. #sc-68343, 1∶750), goat polyclonal anti-fidgetin like 1 (FIGNL1) (C-12) (Cat. #sc-138278, 1∶1000), goat polyclonal anti-FIGNL2 (G-14) (Cat. #sc-242820, 1∶800), mouse monoclonal anti-phosphorylated eukaryotic elongation factor-2 kinase (p-eEF2K) antibody (H-2) (Cat. #sc-377536, 1∶1000), and rabbit polyclonal anti-p-aurora B (phospho T232) (Cat. #ab115793, 1∶500) antibodies were purchased from Santa Cruz (St. Louis, MO, USA). Mouse monoclonal anti-spastin (Cat. #S7074, 1∶1000) and mouse monoclonal anti-β-actin (Cat. #A5316-100, 1∶500) antibodies were purchased from Sigma. Human anti-centromere CREST antibody (Cat. #15-234, 1∶200) was purchased from Antibodies Incorporated (Davis, CA, USA). Rabbit polyclonal anti-eEF2K (Cat. #13510-1-AP, 1∶1000) antibody was purchased from Proteintech (Chicago, IL, USA).

Secondary antibodies of Cy2-conjugated donkey anti-mouse IgG (Cat. #715-225-150, 1∶750), Cy2-conjugated donkey anti-rabbit IgG (Cat. #711-225-152, 1∶500), Rhodamine (TRITC)-conjugated donkey anti-mouse IgG (Cat. #715-025-150, 1∶750), TRITC-conjugated donkey anti-rabbit IgG (Cat. #711-025-152, 1∶500), TRITC-conjugated donkey anti-human IgG (Cat. #709-025-149, 1∶1000), TRITC-conjugated donkey anti-goat IgG (Cat. #705-025-147, 1∶750), and Alexa Fluor 647-conjugated donkey anti-human IgG (Cat. #709-605-149, 1∶500) were purchased from Jackson ImmunoResearch Laboratory (West Grove, PA, USA). Horseradish peroxidase (HRP)-conjugated goat anti-rabbit IgG (Cat. #RA1008, 1∶1000) and HRP-conjugated goat anti-mouse IgG (Cat. #RA1009, 1∶1000) were purchased from Vazyme (Nanjing, China).

eEF2K inhibitor A-484954 (Cat. #324516-10MG,1∶1000), aurora B inhibitor Barasertib (A2D1152-HQPA) (Cat. #S1147, 1∶1000), and ERK 1/2 inhibitor (Cat. #SCH772984, 1∶200) were purchased from Selleckchem (Houston, TX, USA). Nocodazole (M1404) was purchased from Sigma.

### Oocyte collection and culture

Immature oocytes ( *i.e.*, germinal vesicle [GV] oocytes) were obtained from the ovaries of ICR female mice aged three to four weeks. The ovaries were isolated and placed in a surgical medium (Hepes) containing 2.5 nmol/L milrinone and 10% fetal bovine serum (Cat #15260037, Thermo Fisher, Rockford, lL, USA). The follicle was pierced with a hypodermic needle to release the oocyte from the ovary. Cumulus cells were washed from the cumulus-oocyte complex, and each of the 50 isolated molted oocytes was placed in a petri dish (Becton Dickinson, Franklin Lakes, NJ, USA) containing a 100 μL drop of mineral oil. The medium was MEM+ (MEM supplemented with 0.01 mmol/L EDTA, 0.23 mmol/L NA-pyruvate, 0.2 mmol/L pen/strep, 3 mg/mL BSA and 20% fetal bovine serum). Oocytes were cultured in a humidified environment of 37.0 ℃, 5% O _2_, and 5% CO _2_. Before *in vitro* maturation (IVM), all media contain 2.5 nmol/L milrinone to prevent the resumption of meiosis.

### Granulosa cell isolation

Granulosa cell isolation was performed during oocyte collection. When the cumulus-oocyte complexes were first released from the antral follicles within the ovary, cumulus cells were washed off the oocytes and then collected. At this time point, cumulus cells were in a collection buffer with large ovarian tissue fragments. Next, cumulus cells were passed through a cell sieve with a proper pore size to separate them from tissue fragments. Finally, the cumulus cells were resuspended and then spun down at 100 rpm for 5 min, and small debris in the solution was discarded to obtain pure cumulus cells.

### siRNA production and transfection

All DNA template sequences for siRNA production are listed in ***Supplementary Table 1*** (available online). The template sequence for the control siRNA was a simulated sequence that binds nonspecifically to any mRNA from the mouse genome. Four different untranslated regions (UTR) of the *KL2* DNA template, with some modifications, were used for siRNA design through the BLOCK-iT RNAi Designer. The sequence specificity was verified by a BLAST homology search.

siRNA was produced using the T7 RiboMAX ^TM^ Express RNAi system (Promega) according to the manufacturer's instructions. For each double-stranded siRNA that targets one of the four UTRs of *KL2*, two complementary single-stranded siRNAs were synthesized from each of these templates and then annealed to form the final double-stranded siRNA. Next, the siRNA was purified by conventional isopropyl alcohol precipitation. The resulting siRNA mixture was prepared by combining the siRNAs in the same molar ratio at a final concentration of 5 µmol/L. siRNA transfection was performed using the N-TERTM Nanoparticle System (Sigma). The siRNA-nanoparticle complex solution was added to a culture medium containing oocytes and incubated at room temperature. After 12 to 14 h of treatment, the oocytes were rinsed to remove the nanoparticle-containing media. Another round or two of siRNA treatment was performed, depending on how easy it was for the target to be significantly knocked out. Throughout the siRNA treatment, 2.5 nmol/L milrinone (Sigma) was added to prevent the resumption of meiosis. Next, oocytes were transferred to milrinone-free MEM+ medium and cultured for 8 or 16 h.

### Semi-quantitative PCR (sq-PCR)

Oocytes were homogenized in Trizol (Invitrogen, Rockford, lL, USA) and stored at −80 ℃ until further processing. Next, chloroform was used to separate the RNA phase and isopropanol to precipitate the RNA. Afterwards, the suspension was washed with 70% ethanol and the final pellet was resuspended in ultra-pure DEPC-treated water. RNA concentration and quality were measured using a NanoDrop 2000c spectrophotometer (Thermo Fisher Scientific, Rockford, lL, USA). The reverse transcription of the obtained RNA material was performed with SuperScript Ⅲ First-Strand Synthesis System (Invitrogen) and a mixture of random hexanucleotides (Invitrogen) according to the manufacturer's protocol. sq-PCR for the designed oligonucleotides listed in ***Supplementary Table 2*** (available online) was performed using the HiScript Ⅱ One Step RT-PCR Kit (Vazyme) on the GeneExplorer (Bioer Technology, Hangzhou, China). The housekeeping gene glyceraldehyde-3-phosphate dehydrogenase (GAPDH) was used as the internal control for all samples.

### Immunofluorescence (IF) staining

The oocytes were briefly washed in PBS containing 0.05% polyvinylpyrrolidone (PVP), permeabilized with 0.5% Triton X-100/PHEM (60 mmol/L PIPES, 25 mmol/L Hepes pH 6.9, 10 mmol/L EGTA, and 8 mmol/L MgSO _4_) for 5 min, and quickly washed three times in PBS/PVP. The oocytes were then fixed in 3.7% paraformaldehyde (PFA)/PHEM for 20 min, washed in PBS/PVP three times (10 min each), and blocked with blocking buffer (1% BSA/PHEM plus 100 mmol/L glycine) at room temperature for 1 h. The primary antibody was then diluted with blocking buffer, incubated at 4 ℃ overnight, and washed in PBS plus 0.05% Tween-20 (PBST) three times (10 min each time). The primary antibody dilution is as follows: anti-TRIM75, 1∶2000; anti-tubulin, 1∶500; and anti-human centromere, 1∶500. The secondary antibody was diluted with blocking buffer at room temperature for 45 min (1∶750 in all cases) and washed with PBST three times (10 min each time). Finally, the DNA was stained with 10 µg/mL Hoechst 33258 (Sigma), and the oocytes were placed on a slide with a sliding medium (0.5% propyl gallate, 0.1 mol/L Tris-HCl, PH 7.4, and 88% glycerol). The cover glass was 0.13 to 0.17-µm thick. To maintain the size of the oocytes, two 90-µm thick pieces of double-stick tape were placed between the slide and the cover slide. Oocytes were tested with an Andor Revolution confocal workstation (Oxford Instruments, Belfast, Northern Ireland).

### Immunoprecipitation (IP)

In immunoprecipitation experiments, 5 μg control IgG or anti-KL2 antibody was first coupled to a 30 μL protein-A/G agarose bead (Cat #IR005, Macgene, Beijing, China) in 250 μL IP buffer (20 mmol/L Tris-HCl pH 8.0, 10 mmol/L EDTA, 1 mmol/L EGTA, 150 mmol/L NaCl, 0.05% Triton X-100, and 0.05% Nonidet P-40), 1 mmol/L phenyl methyl sulfonyl fluoride) with 1∶100 protease inhibitor (Sigma), and 1∶500 phosphatase inhibitor (Sigma) on a rotating wheel at 4 ℃ for 4 h. Simultaneously, 600 zona pellucida-free GV oocytes were lysed and ultra-sounded in a 250 IP buffer and then pre-cleaned at 4 ℃ with 30 μL protein A/G microbeads for 4 h. Protein A/G coupled control IgG or anti-KL2 antibody was incubated overnight at 4 ℃ with 250 μL pre-cleared oocyte lysate supernatant. Finally, on the following day, the bead was washed three times with 1 mL IP buffer for 10 min each time for subsequent SDS-PAGE and silver staining.

### Silver staining and characterization of KL2-interacting proteins

During silver staining, the immunocomplex beads of the control IgG or anti-KL2 antibody group were boiled in protein sample buffer, the supernatant was separated on SDS-PAGE gel, and the gel was fixed in 4 ℃ fixing solution (10% acetic acid and 40% ethanol) overnight, and then treated at room temperature with freshly prepared sensitizing solution (30% ethanol, 0.2% Na _2_S _2_O _3_, 0.314% Na _2_S _2_O _3_·5H _2_O, and 6.8% sodium acetate) for 30 min, and washed with water three times for 5 min each time. Then, the staining solution (0.25% AgNO _3_ and 0.02% fresh 37% formaldehyde solution) prepared at room temperature was dyed for 20 min, washed for 2.5 min, and placed in the developer solution (2.5% NaCO _3_, 0.02% fresh 37% formaldehyde solution) for approximately 5 to 10 min (depending on the speed of the process). We avoided insufficient or excessive development and finally stopped the development reaction in the stop solution (0.4% glycine) for 10 min.

To identify KL2 interacting protein, the silver staining control and KL2 bands were carefully compared, and the bands with significantly higher gray values were removed one by one and stored in a protease-free tube with 10% ethanol. The bands of the selected possible KL2 interactors were then sent to the Test and Analysis Center of Nanjing Medical University for matrix-assisted laser desorption/ionization time-of-flight mass spectrometry (MALDI-TOF-MS) analysis. We conducted a peptide mass fingerprinting (PMF) search in Mascot for identification.

### Identification of KL2 phosphorylation site and generation of antibodies specific to p-KL2

In general, about 0.01%–0.1% of the protein is phosphorylated, so it is almost impossible to identify the phosphorylation site with oocytes. Therefore, we replaced oocytes with NIH3T3 cells and then produced phosphorylated specific antibodies and validated the antibodies in oocytes. Because of the relatively low abundance of KL2, we used 50 IP reactions, each using 1 × 10 ^6^ NIH3T3 cells, 30 µL protein A/G beads, and 5 µg KL2 antibodies. The immune complex beads were eluted with 0.2 mol/L glycine (PH 2.7), and the phosphorylated portion of the immune complex was enriched with a Pierce TiO _2_ Phosphatide Enrichment and Clean-up Kit (Thermo Scientific). It was sent to the Detection and Analysis Center of Nanjing Medical University for liquid chromatography-mass spectrometry (LC-MS). The proteins were identified by PMF. We selected Tyr5 (85.6% phosphorylation potential) and Thr7 (95.2% phosphorylation potential), and the entire antibody production and purification process was completed by Zhongding Biotechnology Co., Ltd. (Nanjing, Jiangsu, China). The short peptide LS(PTyr)Q(PThr)LKLTHQAC (the "C" on the C term is an additional conjugate residue) was synthesized and injected into rabbits for serum production. Phosphorylated specific antibodies were purified from serum using a phosphopeptide-coupled resin column, and then the residual non-phosphorylated specific antibodies were removed by adsorption using a non-phosphopeptide-coupled resin column (LSYQTLKLTHQAC).

### Live imaging of MT depolymerization and recovery

We used the M16 medium (Sigma) to perform live imaging. The most challenging obstacle for oocyte live imaging is that oocytes in the M16 medium are unable to adhere securely to the glass bottom of the live imaging dish because of the presence of BSA in M16. According to our experience, BSA-free CZB, a widely-used embryo culture medium, can effectively immobilize oocytes; however, because of the need to rapidly cleanse the oocytes to eliminate nocodazole and promote the growth of MTs, the oocytes were distorted and severely damaged when tested in BSA-free CZB medium. We were only able to concentrate on one oocyte at a time, and after taking nocodazole, the quick wash-refocus procedure could be completed in 2 min. Oocytes treated with either control or *KL2* siRNA were subsequently administered with mRNAs of EGFP-tubulin and mRFP-histone injection, followed by an additional 6 to 8 h of incubation in MEM+ medium with milrinone to express exogenous tubulin and histone. Imaging lasted for 2 min, with a Z-step of 0.5 μm and a maximum of four Z-slices per time point. ProLong Live Antifade Reagent (ThermoFisher) was incorporated into M16 at a ratio of 1∶100 to reduce photodamage. Filming continued until the fluorescence intensity of the MTs remained constant.

### Statistical analysis

All experiments were repeated at least three times. Measurement of confocal images was performed using Image J. Data were presented as mean ± standard error of the mean. Statistical comparisons between the two groups were performed with the Student's *t*-test in Excel (Microsoft, Redmond, WA, USA). Multiple comparisons were made using the Kruskal-Wallis one-way nonparametric ANOVA. *P* < 0.05 was considered statistically significant.

## Results

### KL2 was a unique MTSE correlated with chromosome-based spindle organization in mouse oocytes

**Figure 1:**
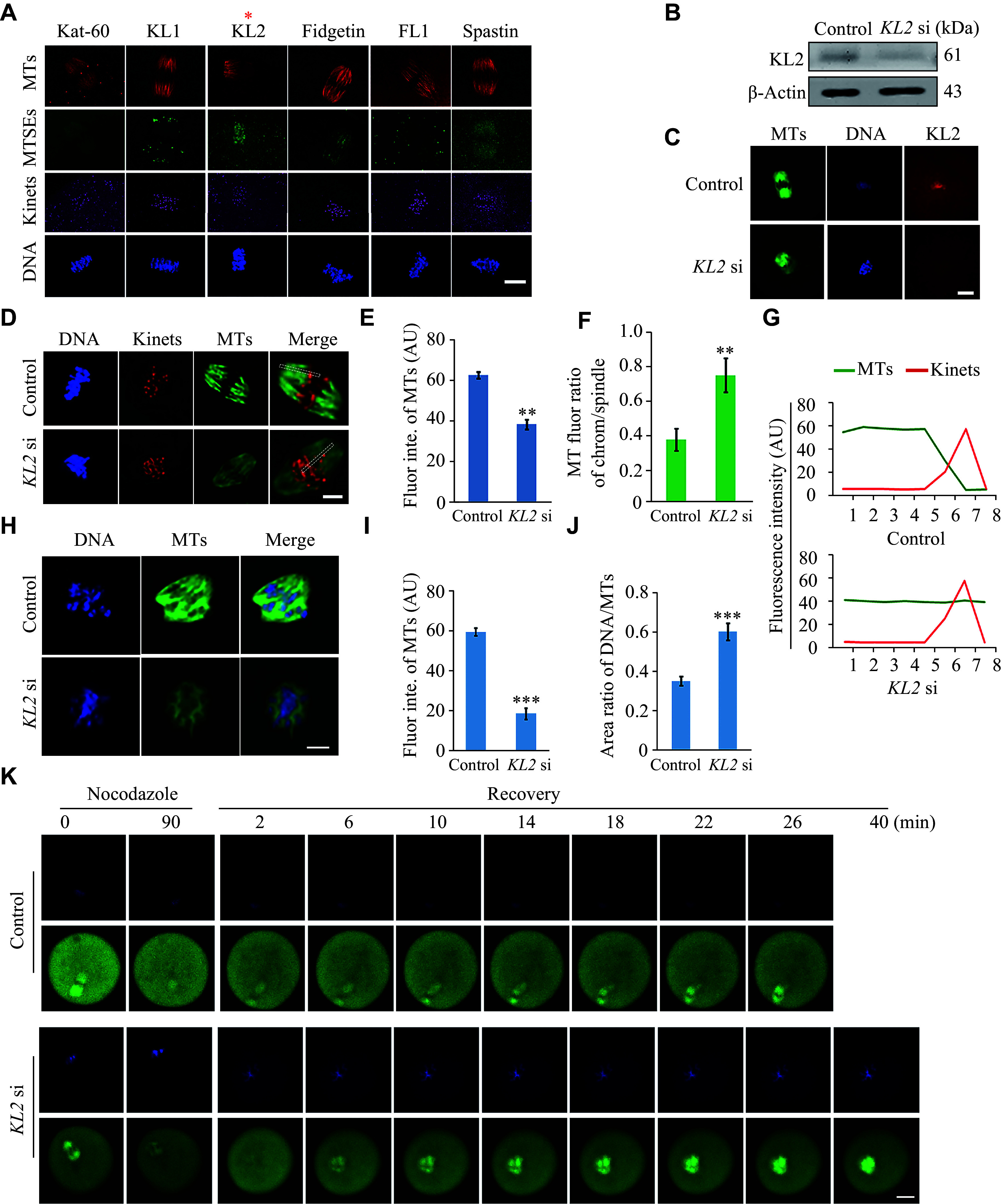
Mouse katanin-like 2 (KL2) was a unique microtubule severing enzyme (MTSE) associated with chromosome-based spindle organization in mouse oocytes.

Seven MTSEs have been identified in mice ^[ 40] ^; however, their functions during meiosis remain unknown. In the current study, we checked their localization patterns during meiosis in mouse oocytes and found that only KL2 was localized to the chromosomes of metaphase Ⅰ (MⅠ) oocytes ( ***Fig. 1A***). Thus, we hypothesized that KL2 might be important for chromosome-based spindle organization.

Subsequently, we knocked down *KL2* expression using *KL2*-specific siRNAs. Both Western blotting and IF staining of KL2 demonstrated that KL2 protein levels were diminished to approximately 30% of those in control oocytes ( ***Fig. 1B*** and **1C **). Moreover, sq-PCR results showed that mRNA levels of other MTSEs were unchanged, indicating the absence of off-target effects ( ***Supplementary Fig. 1A*** and ***1B***, available online).

However, *KL2* knockdown did not significantly affect spindle length or width in MⅠ oocytes, but led to a significant decrease in the MT intensity within the spindle, as demonstrated by the comparison between the control and *KL2*-knockdown (KD) MⅠ oocytes (61.46 *vs.* 39.52 in arbitrary units, respectively) ( ***Fig. 1D*** and ***1E ***). The analysis of kinetochore-MT (K-MT) connections within chromosomes showed that, in *KL2*-KD MⅠ oocytes, the MT plus ends did not correctly connect to outer kinetochores, but rather appeared between kinetochores ( ***Fig. 1D ***). Additionally, the ratio of MT intensity within the chromosome region to that within spindles was significantly elevated in *KL2*-KD MⅠ oocytes than in the control MⅠ oocytes (0.405 *vs.* 0.816) ( ***Fig. 1D*** and ***1F ***). This phenotype was more intuitively shown by drawing a line from the spindle pole to the middle of the chromosome region along the spindle's long axis and continuously measuring the intensity of MTs or kinetochores ( ***Fig. 1G ***). In control MⅠ oocytes, once the kinetochore signal significantly increased, the MT intensity dramatically decreased, while in *KL2*-KD MⅠ oocytes, the MT intensity remained high when the kinetochore signal significantly increased ( ***Fig. 1G ***). These results indicate that *KL2* knockdown may cause MT instability and disrupt normal K-MT attachment.

We further depolymerized MTs in MⅠ oocytes using nocodazole and assessed the MT regrowth process approximately 15 min after the MT recovery. Compared with the control MⅠ oocytes, the MT intensity in *KL2*-KD MⅠ oocytes was significantly low, and the MTs formed an aster-like structure with a small MT area ( ***Fig. 1H ***– ***1J ***). The MTs in control MⅠ oocytes were significantly brighter than those in *KL2*-KD MⅠ oocytes and organized into a pro-MⅠ spindle with clearly distinguishable poles ( ***Fig. 1H ***– ***1J ***).

To observe the whole process of MT depolymerization by nocodazole treatment, we microinjected mRNAs of EGFP-tubulin and mRFP-histone into the control or *KL2*-KD GV oocytes and cultured them for 8 h. Then, MⅠ oocytes underwent live imaging. Although there was no significant difference in the MT depolymerization process, the process of MT regrowth was significantly different between groups. In 55.6% of (5 of 9) the control oocytes, MTs formed well-shaped spindles, and chromosomes kept well congressed at the equator. In the other 44.4% of control oocytes, although chromosomes were less congressed, and the MT intensity in the chromosome region was relatively higher, the MTs still reorganized into well-shaped spindles. However, in 100% (6 of 6) of *KL2*-KD oocytes, MTs did not reorganize into a bipolar spindle for 40 min. The chromosomes were abruptly scattered at the initial recovery stage and were unable to re-congress at the equator ( ***Fig. 1K*** and ***Supplementary Movie 1*** [available online]). These results indicate that KL2 plays a crucial role in the increase of chromosome-based MT polymer and the correction of aberrant K-MT attachment.

### KL2 was essential for normal meiosis progression and fertility in mouse oocytes

**Figure 2:**
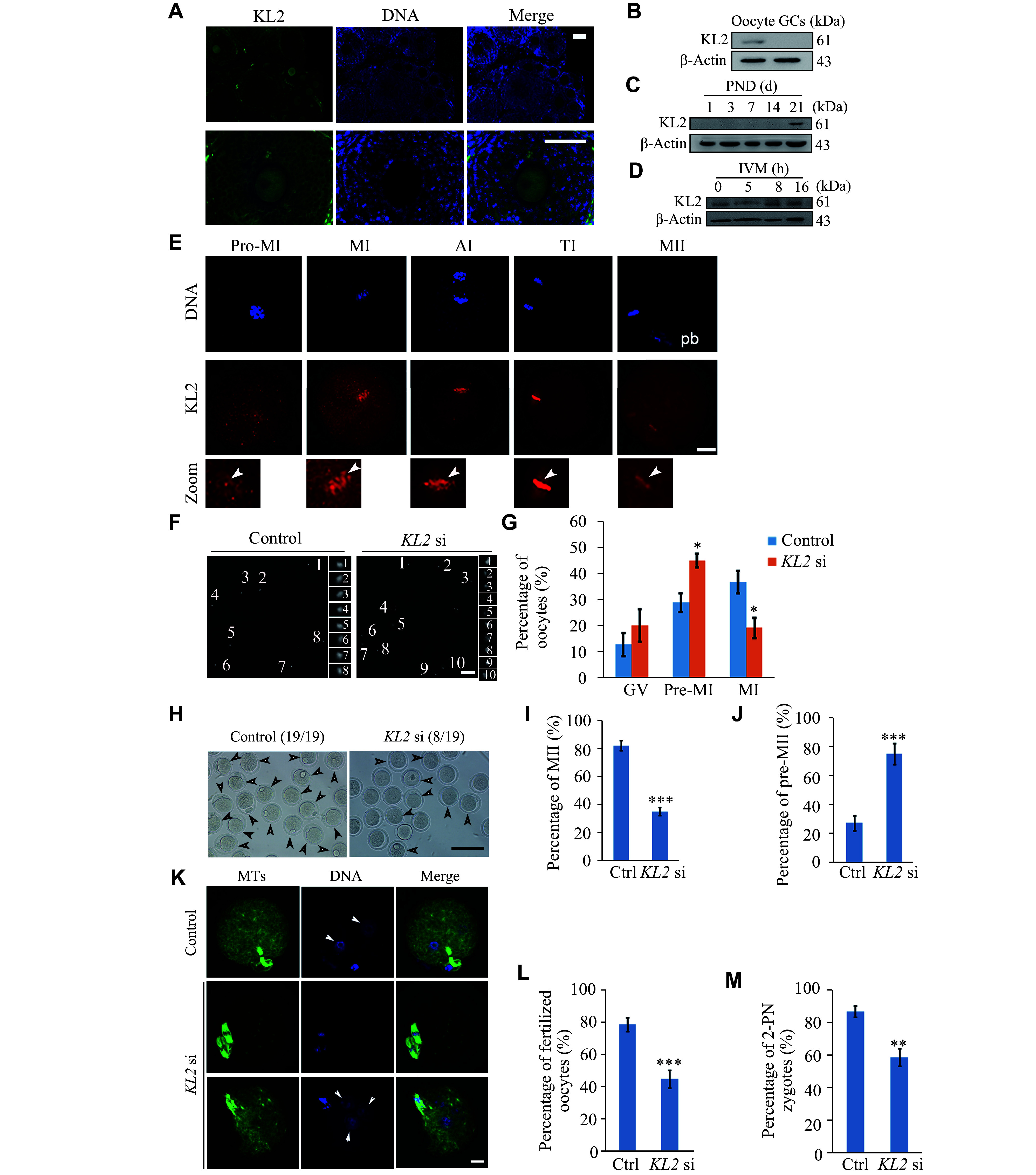
KL2 was important for normal meiosis progression and fertility in mouse oocytes.

Considering the effects of *KL2* knockdown on spindle organization, we next explored whether KL2 was important for normal meiosis and fertility. First, we examined the expression profile of KL2 in ovaries and oocytes. KL2 was much more predominant in oocytes than in granular cells, suggesting that its function is largely isolated to oocytes ( ***Fig. 2A*** and ***2B***). KL2 expression levels within the ovary increased at post-natal day 21 (the first wave of follicle maturation), indicating its association with follicle maturation ( ***Fig. 2C***). During oocyte meiosis, KL2 was localized to the chromosome at the pro-MⅠ, MⅠ, and MⅡ stages but not during AⅠ (anaphase Ⅰ) or TⅠ (telophase Ⅰ) stages, indicating that KL2 was particularly important for spindle organization ( ***Fig. 2D*** and ***2E***).

Next, we cultured GV oocytes *in vitro* for 8 or 16 h. The knockdown of *KL2* significantly reduced the proportion of MⅠ oocytes at 8 h (20.5% *vs.* 38.7%) ( ***Fig. 2F*** and ***2G***) and the proportion of 1pb (first polar body) oocytes at 16 h, compared with the control group (36.2% *vs.* 80.7%) ( ***Fig. 2H*** and ***2I***), indicating a significantly delayed meiotic progression because of *KL2* knockdown. Specifically, the *KL2*-KD group showed a high proportion of oocytes with non-congressed chromosomes, compared with the control group (77.6% *vs.* 29.6%) ( ***Fig. 2J***). These MⅡ oocytes were classified as pre-MⅡ oocytes. We then hypothesized that the non-congressed chromosomes might produce false pronuclei during *in vitro* fertilization. The *KL2*-KD group demonstrated a significantly lower number of fertilized eggs with two pronucleus (2-PN), compared with the control group (59.4% *vs.* 88.8%) ( ***Fig. 2K*** and ***2M***), and also showed a decreased fertility rate (49.6% *vs.* 78.5%) ( ***Fig. 2K*** and ***2L***).

### Aurora B was indispensable for KL2 localization to the chromosomes in mouse oocytes

**Figure 3:**
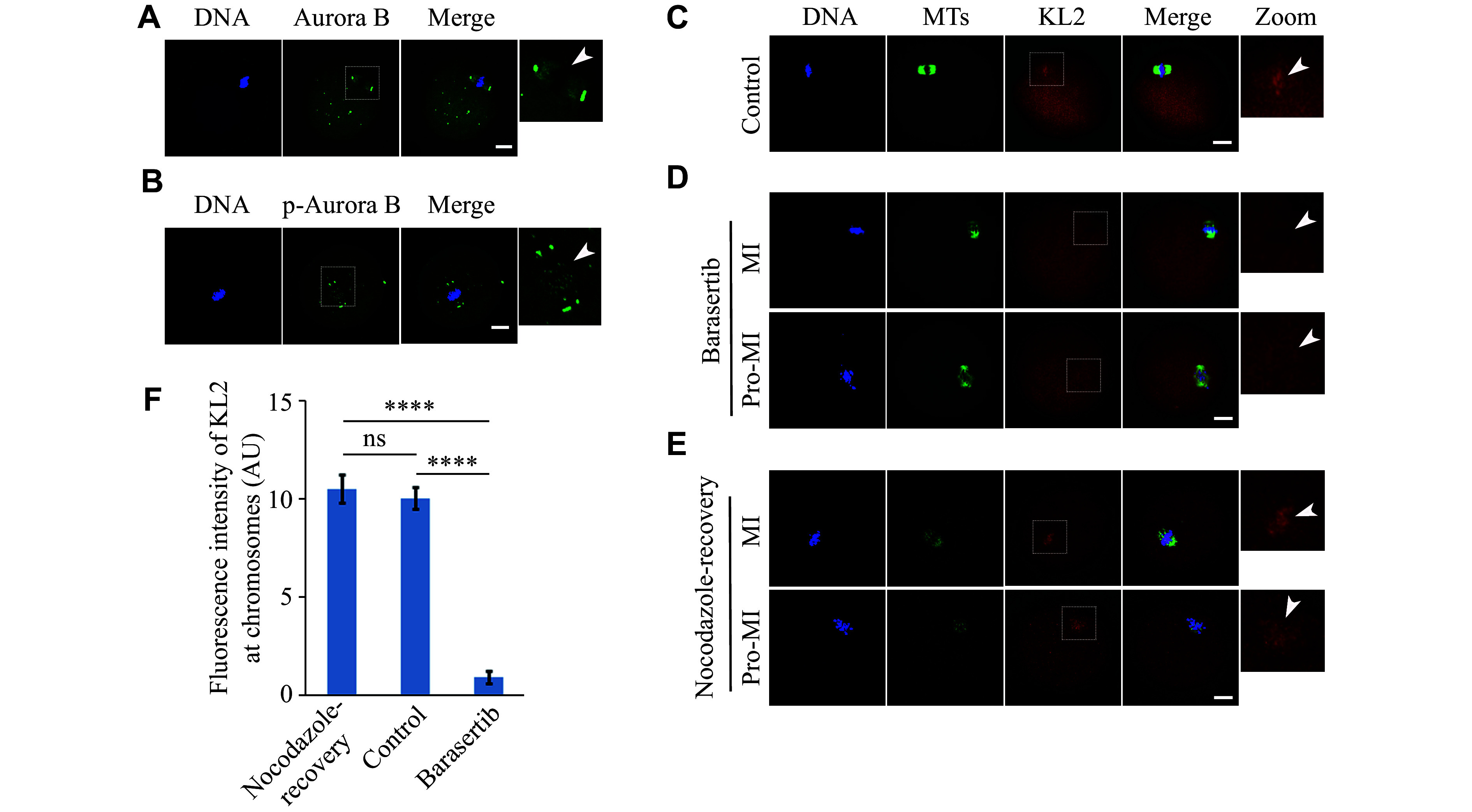
Aurora B was indispensable for KL2 localization to the chromosome in mouse oocytes.

Considering the chromosomal localization of KL2 and the dysfunctional K-MT attachment after *KL2* knockdown, we investigated whether aurora B, a well-known kinase that corrects aberrant K-MT attachment, was important for the KL2 localization. IF staining showed that total aurora B was localized to spindle poles alongside MTs, but it was excluded from chromosomes. In contrast, the phosphorylated aurora B (p-aurora B) was observed as separate dots at chromosomes, but not at spindle poles, indicating that p-aurora B was the active form of aurora B ( ***Fig. 3A*** and ***3B***).

Next, we inactivated aurora B phosphorylation using a specific inhibitor barasertib, and examined the localization of KL2. Consistent with previous studies ^[ 41] ^, the inhibition of aurora B phosphorylation induced aberrant K-MT attachment, and resulted in significantly less congressed chromosomes in the majority of meiosis I oocytes 8 h after *in vitro* maturation ( ***Fig. 3C*** and ***3D***). In these oocytes, KL2 localization to the chromosomes was significantly abolished ( ***Fig. 3D*** [lower] and ***3F***). To exclude the possibility that chromosome scattering, rather than aurora B inactivation, prevented the KL2 localization, we initially demonstrated that KL2 remained absent from the chromosome in the remaining 18.18% of aurora B-inhibited MⅠ oocytes with well-congressed chromosomes ( ***Fig. 3D*** [upper]). Furthermore, in 69.35% of the oocytes that underwent MT depolymerization by nocodazole followed by recovery, the chromosomes were scattered, and the spindle was disorganized; however, the localization of KL2 to the chromosomes did not significantly change ( ***Fig. 3E***). These findings further support that aurora B inhibition led to the KL2 elimination from the chromosome.

### KL2 localization to the chromosomes was modulated by p-eEF2K in mouse oocytes

**Figure 4:**
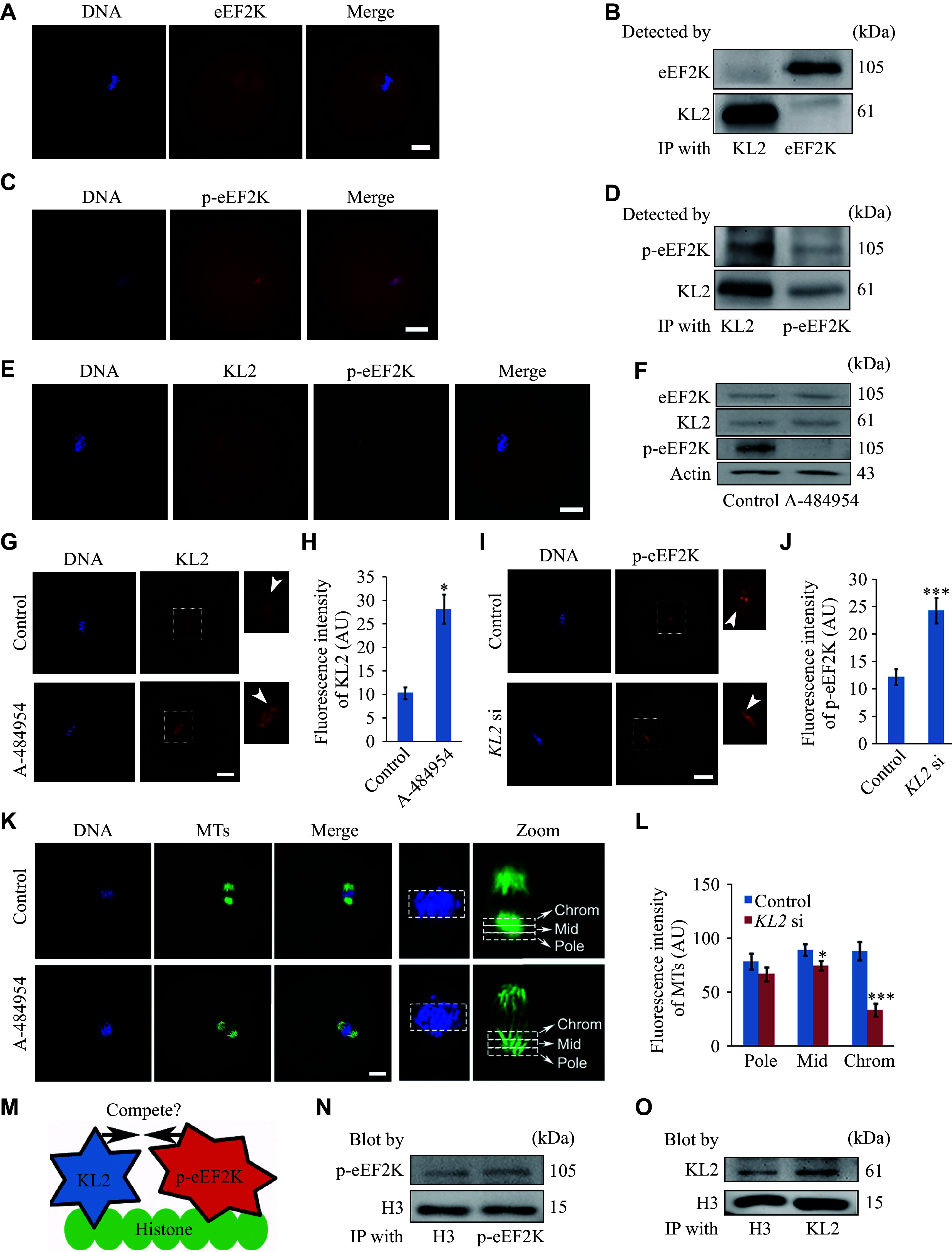
KL2 localization to the chromosomes was modulated by phosphorylated eukaryotic elongation factor-2 kinase (p-eEF2K) in mouse oocytes.

To further investigate the functions of KL2 and how its activity is regulated, we used the KL2 antibody for IP of the MⅠ mouse oocyte lysates. Subsequently, we performed silver staining and identified the bands that were distinct from control IgG by MALDI-TOF-MS. Repeated identification revealed eEF2K as an interacting protein ( ***Supplementary Fig. 2A*** and ***2B*** [dot-line labeled], available online). Following this, we conducted tests to examine the localization and phosphorylation of eEF2K during meiosis. Our findings revealed that eEF2K was enriched in the spindle region but was excluded from chromosomal regions ( ***Fig. 4A***), whereas p-eEF2K was highly localized to the chromosome ( ***Fig.4C*** and ***4E***). Co-IP results showed that KL2 interacted with p-eEF2K but less so than with eEF2K, which is consistent with the localization patterns of these proteins ( ***Fig. 4B*** and ***4D***). To further investigate these protein interactions, we reduced p-eEF2K levels using a specific inhibitor ( ***Fig. 4F***) and then performed KL2 staining. The KL2 signal intensity at the chromosome significantly increased ( ***Fig. 4G*** and ***4H***) by more than one-fold. On the other hand, KL2 knockdown significantly increased the localization of p-eEF2K to the chromosome ( ***Fig. 4I*** and ***4J***) by more than one-fold. Furthermore, we partitioned the spindle region beyond the chromosome along the long axis into three equal-height regions (pole, mid, and chrom) and compared the MT intensity of each region between the control and p-eEF2K inhibition groups. Interestingly, we found no significant differences in the pole or mid regions; however, the MT intensity in the chrom region was significantly decreased in p-eEF2K inhibition group than the control group, indicating that the inhibition of p-eEF2K might have affected MTs near the chromosome rather than other spindle regions ( ***Fig. 4K*** and ***4L***).

Based on the results presented above, we hypothesized that KL2 and p-eEF2K might compete for binding to the same chromosome components ( ***Fig. 4M***). Our preliminary LC-MS results showed that KL2 pulled down histone 1 (H1), H2, and H3, and that both KL2 and p-eEF2K interacted with H3 ( ***Fig. 4N*** and ***4O***), H1 ( ***Supplementary Fig. 3A*** and ***3B*** [available online]), and H2 ( ***Supplementary Fig. 3C*** and ***3D*** [available online]).

### ERK 1/2 regulated the relocation of KL2 to spindle poles *via* phosphorylation at Tyr5 and Thr7 in mouse oocytes

**Figure 5:**
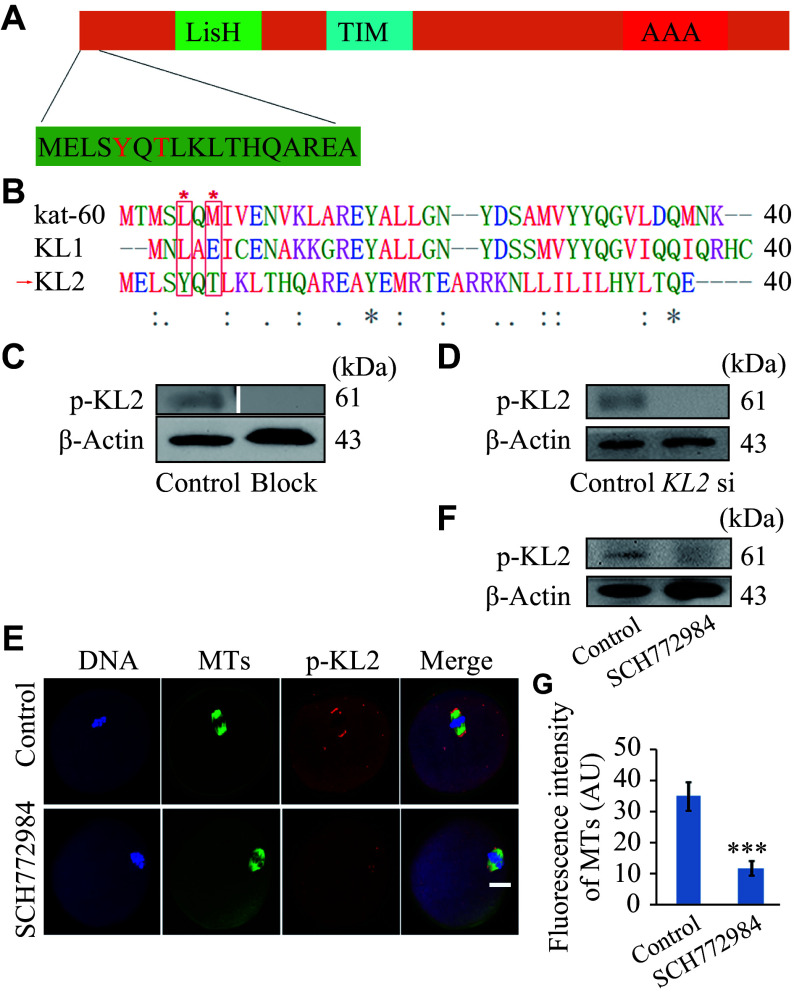
ERK 1/2 regulated the relocation of KL2 to spindle poles *via* phosphorylation at Tyr5 (Y5) and Thr7 (T7) in mouse oocytes.

Finally, we investigated whether the KL2 activity was regulated by phosphorylation, which is the most common method for modulating protein activity. After a phosphopeptide enrichment following IP in NIH3T3 cell lysates, we identified the phosphorylation sites using LC-MS. Two sites of interest were identified as Tyr5 (85.6% phosphorylation possibility) and Thr7 (95.2% phosphorylation possibility) ( ***Fig. 5A***). Notably, these two sites are not conserved even in katanins (such as katanin 60, KL1, and KL2, ***Fig. 5B***). In other words, among all the MTSEs, these sites are unique to KL2.

Subsequently, we generated phospho-specific antibodies against these two sites (p-KL2) and verified their specificity through two approaches. Firstly, an oocyte sample immunoblotted by p-KL2 antibody showed a clear band of an expected size. Conversely, when the p-KL2 antibody was pre-blocked with the KL2 phosphopeptide that was used for p-KL2 antibody generation, no band appeared at the expected position ( ***Fig. 5C***). Secondly, the knockdown of *KL2* efficiently reduced the levels of p-KL2 ( ***Fig. 5D***). IF staining showed that p-KL2 was localized to the spindle poles and existed as separate dots within the cytoplasm, while being excluded from chromosomes ( ***Fig. 5E***). Therefore, it is likely that KL2 becomes inactive after undergoing phosphorylation. Notably, the inhibition of extracellular signal-regulated kinase 1/2 (ERK1/2) with a specific inhibitor SCH772984 significantly reduced the level of p-KL2 ( ***Fig. 5F***) and its localization to the spindle poles ( ***Fig. 5E*** and ***5G***), suggesting that active ERK1/2 is required for the KL2 phosphorylation.

## Discussion

MTSEs may have three distinct roles during the cell cycle: exposing MT ends for successive depolymerization, increasing MT polymer mass at their location, and removing aberrant K-MT attachments. Several studies have demonstrated the first role ^[ 7, 13, 22– 24, 42] ^. In addition, increasing evidence supports the role of MTSEs in increasing MT polymer. In human mitotic U2OS cells, KL1 was localized specifically to the spindle poles, and KL1 knockdown significantly reduced the microtubule intensity at the poles ^[ 43] ^. Thus, MT severing generates MT seeds within spindle poles leading to an increase in the MT polymer. During meiosis in *C. elegans*, MEI-1, a homolog of katanin 60, was localized to chromosomes, and the loss of MEI-1 reduced the MTs terminating at the chromosomes. These findings indicate that MEI-1 is responsible for increasing the MT polymer number at the chromosomes ^[ 44] ^. The current study indicates that KL2 may induce a chromosome-based increase in the MT polymer and thereby play a crucial role in meiotic spindle organization.

There is no direct evidence supporting the role of MTSEs in removing aberrant K-MT attachments. However, MCAK, a kinesin-13 MT depolymerase, was regulated by aurora B and caused the release of improper MT attachments ^[ 17, 45] ^. Depolymerase is thought to function in coordination with MTSEs during both mitosis ^[ 29, 46]^ and interphase ^[ 47] ^. Therefore, based on our findings, it is reasonable to speculate that aurora B may regulate the severing activity of KL2 to remove merotelic attachments and ensure amphilic attachments during mammalian female meiosis.

Our results indicate that KL2 plays a crucial role in female meiosis progression, thereby affecting the quality of matured oocytes (MⅡ oocytes) and normal fertility. Abnormally fertilized eggs may result in abnormal newborns. Although KL2 has not been reported to affect mammalian female meiosis and fertility, katanin p80, which targets katanin 60, is essential for male meiotic spindle assembly and cytokinesis. Mutations in katanin p80 caused male sterility characterized by the decreased sperm production, abnormal head shapes in sperm, and the decreased motility ^[ 39] ^. In more primitive organisms, the function and regulation of katanin in meiosis have been well studied ^[ 3, 9, 25, 48– 50] ^.

EEF2K, also well-known as calmodulin-dependent protein kinase Ⅲ (CaM kinase Ⅲ), is a unique member of the Ca ^2+^/CaM-dependent protein kinase family. The activation of CaM kinase Ⅲ leads to the selective phosphorylation of eukaryotic elongation factor 2 (eEF-2) and transient inhibition of protein synthesis ^[ 51] ^. No studies have reported a specific mitotic or meiotic localization of this protein, nor established its association with spindle organization during meiosis. However, eEF2K is regulated by CDC2-cyclin B, the key regulator of mitosis ^[ 52] ^, indicating its involvement in mitosis. In the current study, we demonstrated that the inhibition of p-eEF2K significantly increased the KL2 level at chromosomes and decreased the MT intensity near the chromosome region, suggesting that KL2 localization to the chromosomes may be modulated to maintain normal spindle organization.

Multiple studies have demonstrated that the activity of MTSEs is regulated by phosphorylation. In *C. elegans*, the katanin homolog MEI-1 is required for meiosis, but it must be inactivated through dephosphorylation by PPFR-1, a regulatory subunit of a trimeric complex of protein phosphatase 4, before the start of mitosis ^[ 53] ^. In *Xenopus* egg extracts, N-terminal phosphorylation of katanin 60 decreased its severing activity and affected spindle length control ^[ 54] ^. Therefore, similar homologs of MTSEs in different species may be regulated distinctly by phosphorylation and dephosphorylation. The regulation of KL2 by phosphorylation and dephosphorylation has not been established; furthermore, our findings indicate that its modulation appears to differ from other MTSEs. ERK1/2 is known to be important in meiosis ^[ 55] ^; however, no studies have demonstrated its role in regulating the activity or chromosomal localization of MTSEs. We did not determine whether ERK1/2 directly or indirectly phosphorylated KL2, but it did produce an upstream signal to trigger this process.

**Figure 6:**
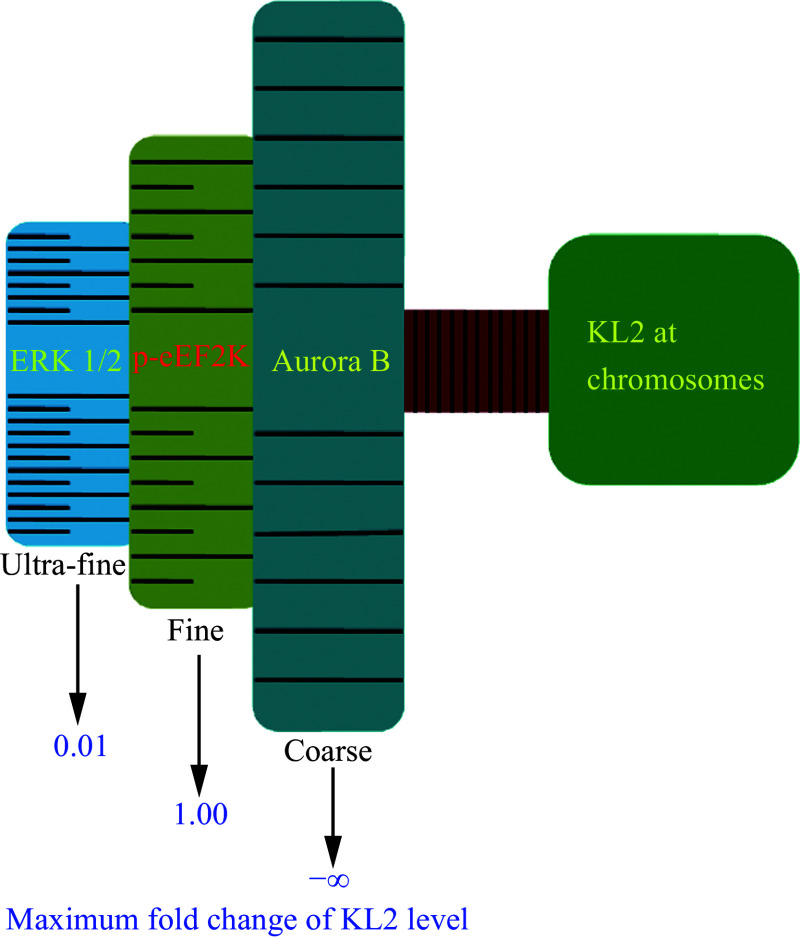
Aurora B, p-eEF2K, and ERK 1/2 coordinate to modulate the chromosomal localization of KL2 in a "multilevel focus-adjusting" manner in mouse oocytes.

Together, aurora B, p-eEF2K, and ERK1/2 collectively modulated the chromosomal localization of KL2 in a "multilevel focus-adjusting" manner ( ***Fig. 6***). Specifically, the activation/inactivation of aurora B causes the presence/absence of KL2 at chromosomes, thus modulating KL2 in a "coarse focusing" manner. Next, the activation/inactivation of p-eEF2K exerts a "fine focusing" modulation of KL2 levels, as evidenced by 1- to 2-fold changes in the expression. Finally, the activation/inactivation of ERK1/2 leads to the phosphorylation/dephosphorylation of a small proportion of KL2 (0.01%–0.1%), representing an "ultra-fine focusing" method.

In summary, we have identified KL2 as a unique member of the MTSE family, which localizes to the chromosome and functions to both increase MT polymer levels and correct aberrant K-MT attachments. Therefore, the current study reveals a novel function of this unique MTSE in female meiosis and demonstrates that multiple kinases coordinate to regulate KL2 levels at chromosomes. Further investigation is needed to explore the regulation of KL2.

## SUPPLEMENTARY DATA

Supplementary data to this article can be found online.
