# Supplementary material for: Mouse KL2 is a unique MTSE involved in chromosome-based spindle organization and regulated by multiple kinases during female meiosis
Source: J Biomed Res. 2024 May 29;38(5):485–99. doi: 10.7555/JBR.37.20230290 (PMC11461529; doi:10.7555/JBR.37.20230290)
Supplement: Supplementary file 1 — Supplementary data to this article can be found online. [file jbr-38-5-485-S1.pdf]

Available online at www.jbr-pub.org.cn

Open Access at PubMed Central

Journal of Biomedical Research, 2024 38(5): 1–3

Supplementary Data

JBR

Mouse KL2 is a unique MTSE involved in chromosome-based spindle organization and regulated by multiple kinases during female meiosis

Shiya Xie[1,2,3,△], Yanjie Yang[1,2,3,△], Zhen Jin[4,5,△], Xiaocong Liu[6,△], Shuping Zhang[1], Ning Su[1], Jiaqi Liu[1], Congrong Li[1], Dong Zhang[1,3,✉], Leilei Gao[4,✉], Zhixia Yang[2,✉]

[1]State Key Lab of Reproductive Medicine and Offspring Health, Nanjing Medical University, Nanjing, Jiangsu 211166, China;
[2]Central Laboratory, the First Affiliated Hospital of Anhui Medical University, Hefei, Anhui 230022, China;
[3]Department of Gynaecology and Obstetrics, the First Affiliated Hospital of Anhui Medical University, Hefei, Anhui 230022, China;
[4]Center for Reproductive Medicine, Department of Gynecology, Zhejiang Provincial People's Hospital (Affiliated People's Hospital), Hangzhou Medical College, Hangzhou, Zhejiang 310014, China;
[5]Center for Reproductive Medicine, Department of Reproductive Endocrinology, Zhejiang Provincial People's Hospital (Affiliated People's Hospital), Hangzhou Medical College, Hangzhou, Zhejiang 310014, China;
[6]Laboratory Department of Shihezi People's Hospital, Shihezi, Xinjiang 832099, China.

Supplementary Movie 1 (available online) shows that the *KL2* knockdown caused disorganized spindle and uncompressed chromosomes during the nocodazole treatment and recovery process (available online). Oocytes were treated with control or *KL2* siRNA treatment, then injected with mRNAs of EGFP-tubulin and mRFP-histone and cultured till 8 h. M I oocytes were first treated with nocodazole and quickly subjected to filming at 10 min intervals per frame. After 90 min, the microtubules were almost completely disassembled. Then oocytes were quickly washed to remove nocodazole and subjected to filming for microtubule re-growth at 2 min intervals per frame.

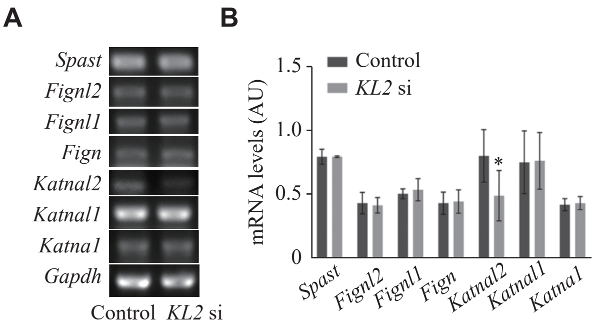

***Supplementary Fig. 1 KL2* siRNA treatment specifically reduced *KL2* mRNA levels.** A and B: Semi-quantitative PCR analyses were performed to compare the mRNA levels of each microtubule-severing protein (*i.e.*, spastin [*Spast*], fidgetin-like 2 [*Fignl2*], fidgetin-like 1 [*Fignl1*]), fidgetin [*Fign*], katanin-like 2 [*Katnal2*], katanin-like 1 [*Katnal1*], and katanin [*Katna1*], between the control and *KL2* siRNA (*KL2* si) treatment oocytes. Data are shown as mean ± standard error of the mean (n=3). Student's t-test was used for statistics between two groups. $^{*}P < 0.05$.

△These authors contributed equally to this work.

✉Corresponding authors: Dong Zhang, State Key Lab of Reproductive Medicine and Offspring Health, Nanjing Medical University, 101 Longmian Ave., Nanjing, Jiangsu 211166, China. E-mail: dong.ray.zhang@njmu.edu.cn; Leilei Gao, Center for Reproductive Medicine, Department of Gynecology, Zhejiang Provincial People's Hospital (Affiliated People's Hospital), Hangzhou Medical College, 158 Shangtang Road, Hangzhou, Zhejiang 310014, China. E-mail: gaoleilei198802@126.com; ZhiXia Yang, Central Laboratory, the First Affiliated Hospital of Anhui Medical University, 218 Jixi Road, Hefei, Anhui 230022, China. E-mail: yang_zhixia@sina.com.

Received: 01 December 2023; Revised: 03 March 2024; Accepted: 05 March 2024; Published online: 29 May 2024

CLC number: R321.1, Document code: A

The authors reported no conflict of interests.

This is an open access article under the Creative Commons Attribution (CC BY 4.0) license, which permits others to distribute, remix, adapt and build upon this work, for commercial use, provided the original work is properly cited.

© 2024 by Journal of Biomedical Research.

https://doi.org/10.7555/JBR.37.20230290

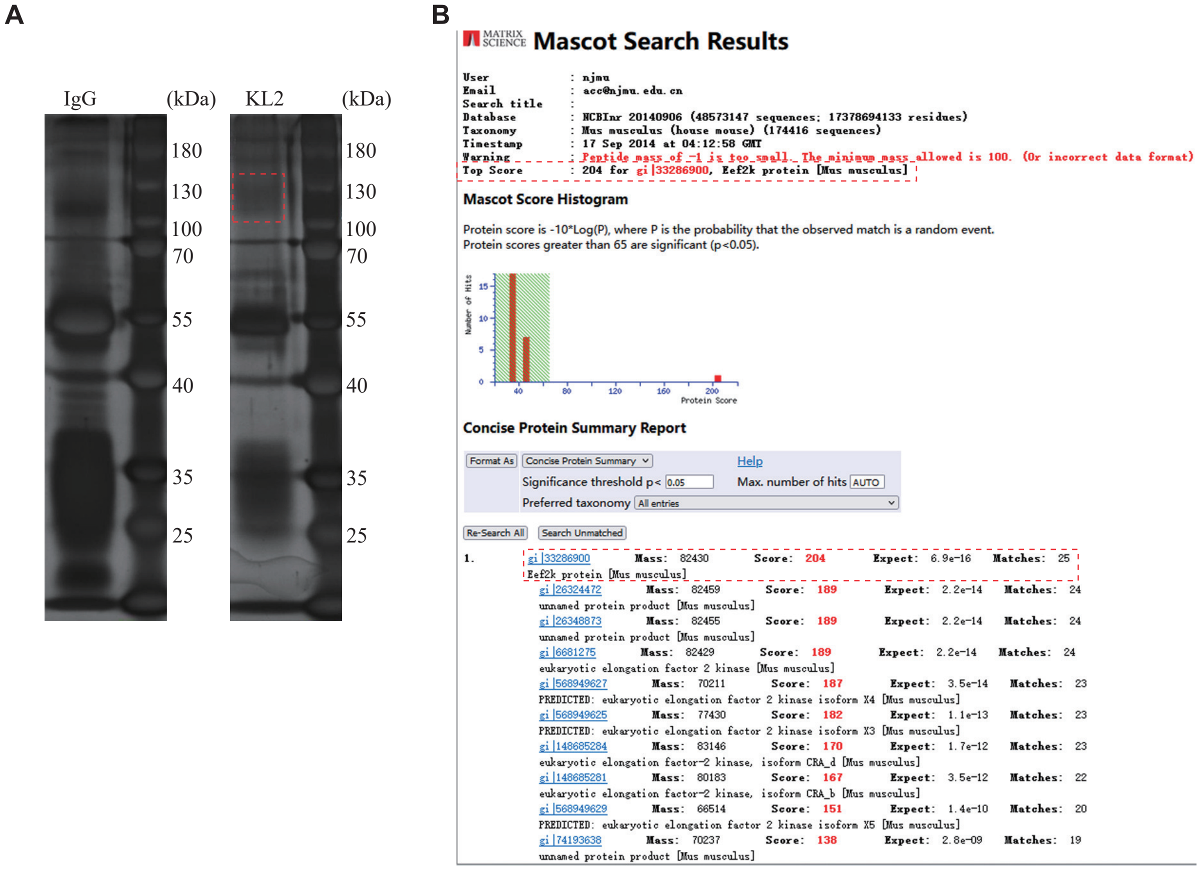

Supplementary Fig. 2 **LC-MS identification of immunoprecipitation with anti-KL2 antibody in oocyte lysate** A: Immunoprecipitation with control IgG or anti-KL2 antibody in oocyte lysate, SDS-PAGE, and silver staining were performed to identify probable KL2 interacting proteins. The predictable gel regions were cut and sent for liquid chromatography-mass spectrometry (LC-MS) identification. B: Mascot Search was used to identify the proteins within the gel regions.

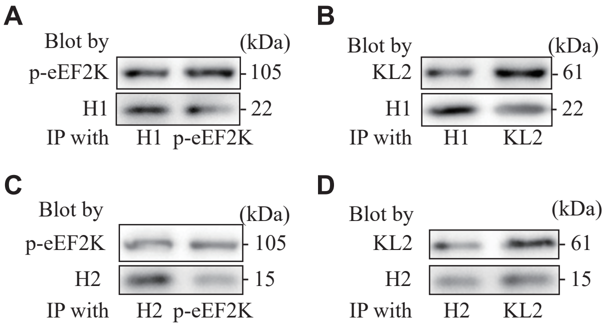

Supplementary Fig. 3 **Both p-eEF2K and KL2 interacted with histone 1 and 2.** A and B: Co-immunoprecipitation (Co-IP) and Western blotting showed the interaction between p-eEF2K and H1 (A) or between KL2 and H1 (B). C and D: Co-IP and Western blotting showed the interaction between p-eEF2K and H2 (C) or between KL2 and H2 (D).

Supplementary Table 1 **DNA oligos for siRNA production**

Target site	DNA templates (5′–3′)
KL2 UTR (4–24)[1]	Oligo 1: GGATCCTAATACGACTCACTATAGACTTTCTCAGAGGGCGTCCA[2]<br>Oligo 2: AATGGACGCCCTCTGAGAAAGTCTATAGTGAGTCGTATTAGGATCC[2]<br>Oligo 3: GGATCCTAATACGACTCACTATATGGACGCCCTCTGAGAAAGTC[2]<br>Oligo 4: AAGACTTTCTCAGAGGGCGTCCATATAGTGAGTCGTATTAGGATCC[2]
KL2 UTR (103–123)[1]	Oligo 1: GGATCCTAATACGACTCACTATAGAGACAGACAGAAAGCACTTC[2]<br>Oligo 2: AAGAAGTGCTTTCTGTCTGTCTCTATAGTGAGTCGTATTAGGATCC[2]<br>Oligo 3: GGATCCTAATACGACTCACTATAGAAGTGCTTTCTGTCTGTCTC[2]<br>Oligo 4: AAGAGACAGACAGAAAGCACTTCTATAGTGAGTCGTATTAGGATCC[2]
KL2 UTR (193–213)[1]	Oligo 1: GGATCCTAATACGACTCACTATAGATAAATACATTCATAATCTA[2]<br>Oligo 2: AATAGATTATGAATGTATTTATCTATAGTGAGTCGTATTAGGATCC[2]<br>Oligo 3: GGATCCTAATACGACTCACTATATAGATTATGAATGTATTTATC[2]<br>Oligo 4: AAGATAAATACATTCATAATCTATATAGTGAGTCGTATTAGGATCC[2]
KL2 UTR (310–330)[1]	Oligo 1: GGATCCTAATACGACTCACTATAGAGTGGATTCCAATAGAGAAT[2]<br>Oligo 2: AAATTCTCTATTGGAATCCACTCTATAGTGAGTCGTATTAGGATCC[2]<br>Oligo 3: GGATCCTAATACGACTCACTATAATTCTCTATTGGAATCCACTC[2]<br>Oligo 4: AAGAGTGGATTCCAATAGAGAATTATAGTGAGTCGTATTAGGATCC[2]
Control[3]	Oligo 1: GGATCCTAATACGACTCACTATACCTACGCCACCAATTTCGTTT[2]<br>Oligo 2: AAAAACGAAATTGGTGGCGTAGGTATAGTGAGTCGTATTAGGATCC[2]<br>Oligo 3: GGATCCTAATACGACTCACTATAAAACGAAATTGGTGGCGTAGG[2]<br>Oligo 4: AACCTACGCCACCAATTTCGTTTTATAGTGAGTCGTATTAGGATCC[2]

[1]The numbers are the starting and ending position of the target sites in *KL2* 3′ untranslated region (UTR) (NM_027721.2 in NCBI).
[2]Two pairs of DNA oligos are needed for each double-stand siRNA. Oligo 2 is complementary with oligo 1 except for an "AA" overhang at 5′; Oligo 3 is complementary with oligo 4 except for an "AA" overhang at 5′. In each oligo, gene-specific sequences are underlined, other sequences are for recognition and binding by T7 RNA polymerase.
[3]Control siRNA does not target any mRNA sequence in mice.

Supplementary Table 2 **RT-PCR primers for microtubule-severing enzymes**

Primer name	DNA templates (5′-3′)
Fign-F	Oligo: TGACATCACCTCGACCACTC
Fign-R	Oligo: GACGCAGTCAGAGCAGATATG
Spast-F	Oligo: ATAGTTACGGGCCAAGGTGAA
Spast-R	Oligo: AAAACTGGTTGCAGCTTCTCT
Katnal1-F	Oligo: ATGAATTTGGCGGAGATTTGTGA
Katnal1-R	Oligo: GTGCTGACGATACTCTTAACCTG
Katnal2-F	Oligo: CACAAGAAGGCTACATGGATGC
Katnal2-R	Oligo: CCTCCACTTCGTGACGGTAAAT
Katna1-F	Oligo: AAATTGGCTCGTGAATATGCACT
Katna1-R	Oligo: CGGAGGTGTGTATCTTTGACTG
Fignl2-F	Oligo: CAGCCCCTAAACCAGTGGC
Fignl2-R	Oligo: GCGGAAATGTCGTCGTGTG
